# Thermal Management of Microelectronic Devices Using Nanofluid with Metal foam Heat Sink

**DOI:** 10.3390/mi14071475

**Published:** 2023-07-23

**Authors:** Muhammad Teham Tahir, Shahzaib Anwar, Naseem Ahmad, Mariyam Sattar, Usama Waleed Qazi, Usman Ghafoor, Muhammad Raheel Bhutta

**Affiliations:** 1 Department of Mechanical Engineering, Institute of Space Technology, Islamabad 44000, Pakistan; tahirteham@gmail.com (M.T.T.); shahzaibanwar792@gmail.com (S.A.); naseem_saddiqui@yahoo.com (N.A.); waleed.kazi@yahoo.com (U.W.Q.); 2 Department of Aeronautics and Astronautics, Institute of Space Technology, Islamabad 44000, Pakistan; mariya98975@gmail.com; 3 School of Mechanical Engineering, Pusan National University, Busan 46241, Republic of Korea; 4 Department of Electrical and Computer Engineering, University of UTAH Asia Campus, Incheon 21985, Republic of Korea

**Keywords:** thermal management, microelectronics, metal foam, nanofluids, numerical simulation

## Abstract

Microelectronic components are used in a variety of applications that range from processing units to smart devices. These components are prone to malfunctions at high temperatures exceeding 373 K in the form of heat dissipation. To resolve this issue, in microelectronic components, a cooling system is required. This issue can be better dealt with by using a combination of metal foam, heat sinks, and nanofluids. This study investigates the effect of using a rectangular-finned heat sink integrated with metal foam between the fins, and different water-based nanofluids as the working fluid for cooling purposes. A 3D numerical model of the metal foam with a BCC-unit cell structure is used. Various parameters are analyzed: temperature, pressure drop, overall heat transfer coefficient, Nusselt number, and flow rate. Fluid flows through the metal foam in a turbulent flow with a Reynold’s number ranging from 2100 to 6500. The optimum fin height, thickness, spacing, and base thickness for the heat sink are analyzed, and for the metal foam, the material, porosity, and pore density are investigated. In addition, the volume fraction, nanoparticle material, and flow rate for the nanofluid is obtained. The results showed that the use of metal foam enhanced the thermal performance of the heat sink, and nanofluids provided better thermal management than pure water. For both cases, a higher Nusselt number, overall heat transfer coefficient, and better temperature reduction is achieved. CuO nanofluid and high-porosity low-pore-density metal foam provided the optimum results, namely a base temperature of 314 K, compared to 341 K, with a pressure drop of 130 Pa. A trade-off was achieved between the temperature reduction and pumping power, as higher concentrations of nanofluid provided better thermal management and resulted in a large pressure drop.

## 1. Introduction

Recent technological developments for the enhancement of the processing capacity of microelectronics and miniaturization have resulted in increased heat dissipation requirements. Over the period of the last three decades, nanofluids have gained immense popularity in the field of heat transfer. Nanoparticles enhance the thermal properties of the base fluid, thereby increasing heat dissipation. Failing to achieve the required heat dissipation affects the performance and ultimately results in the malfunctioning of the device [1]. Elnaggar [2] carried out an analytical investigation and monitored the effect of the number of fins and the fin thickness on the performance of the heat sink. Results showed an increase in the heat transfer rate; however, the effect of the fin number was more significant compared to that of the fin thickness. The reason is a choked flow in heat sink channels due to air compression [3]. Optimum fin spacing leads to the maximum heat transfer coefficient, Nusselt number, and heat transfer rate. It also leads to a decrease in heat sink weight [4]. Boomsma and Poulikakos [5] developed a thermal conductivity model for porous metal foam using tetrakaidecahedrons. This thermal conductivity model estimated the effective thermal conductivity very well for experimental configurations.

Bhattacharya and Mahajan [6] experimentally investigated the use of metal-foam heat sinks in electronics for forced convective heat transfer. A considerable enhancement in heat transfer with the use of metal foam heat sink was observed. Compared to finned heat sinks, metal-foam heat sinks had a uniform surface temperature, fast heat dissipation, and no heat accumulation. With an increase in pour density, the heat transfer coefficient is enhanced [7]. Krishnan et al. [8] conducted research on BCC structures with metal foam, and the results showed that the model predicts the parameters well at high porosities. Lin et al. [9] worked on LTE and LTNE models to investigate thermal performance. Their results revealed that the LTE models predicted as good average Nusselt numbers as the LTNE models. The thermal non-equilibrium effect between the solid and fluid phases is investigated by [10]. Brinkmann–Forchheimer [11] used the CFD approach with aluminum foam, and the results showed that due to the strong flow-mixing capabilities of the foam structures, its velocity profile remains unchanged in the flow direction.

Copper nanofluids and alumina nanofluids with various volume fractions inside silicon microchannel heat sinks were investigated [12,13,14,15] to determine if a reduction in the base temperature would result in a substantial increase in wall shear stress [16]. CuO/water nanofluid was used with different concentrations, and its performance was compared with that of water. The use of nanofluids decreased the thermal stresses and failure rate of electronics chips [17]. An analytical comparison of SiC nanofluid to TiO_2_ showed an increase in thermal conductivity. Moreover, an increase in concentration and flow velocity was observed to increase heat dissipation [18]. A numerical study with the same nanofluids revealed an increase in the heat transfer coefficient, volume fraction, and Reynold’s number [19]. The numerical model validated the analytical results discussed in reference [18]. Another study compared Al_2_O_3_, SiC, and CuO nanofluids at different concentrations and velocities. SiC and CuO nanofluids gave the highest thermal conductivity and heat flux enhancement, respectively. Moreover, the CuO nanofluid was found most suitable for the cooling of electronic devices [20].

Bayomy and Saghir [21] conducted an experimental and numerical investigation of the heat transfer characteristics of heat sinks (with 0, 2, and 3 channels) made of aluminum metal foam subjected to a steady water flow. The results showed a low surface temperature, a high average Nusselt number, and a pressure drop for the heat sink without a channel. Xu et al. [22] applied continuity, energy, and momentum equations to numerically investigate flow and heat transfer characteristics of nanofluids passing through copper metal foam ducts. Nanofluids increased the pressure drop. Moreover, a uniform velocity profile is observed for the large difference in conductivity between the solid and fluid states.

Ghaziani and Hassanipour [23] performed an experimental study with metal foam-filled heat sinks, through which the Al_2_O_3_ nanofluid flowed at different volume concentrations. The heat transfer enhancement using the Al_2_O_3_ nanofluid in porous media was studied under various flow velocities, heat flux, and particle concentrations. A significant improvement in heat transfer was observed when both metal foam and nanofluids were used. However, chaotic movements, dispersions, fluctuations, and interactions with porous media augmented the heat transfer. Pourfarzad et al. [24] experimentally investigated heat transfer and pressure drop at different concentrations of alumina nanofluid at different metal foam pore densities. Better results were obtained at a low pore density and a high-volume fraction of nanofluid. Saghir et al. [25] experimentally analyzed whether heat enhancement is possible with forced convection and nanofluids at high flow rates as compared to water. In a numerical investigation, Al_2_O_3_/water, Al_2_O_3_/ethyl glycol, TiO_2_/water, and TiO_2_/ethyl glycol nanofluids are passed through porous blocks, porous straight channels, and porous wavy channel heat sinks. In all these cases, water proved to be the better base fluid and the Al_2_O_3_ nanofluid showed enhanced heat extraction and temperature uniformity [26]. Welsford et al. carried out an experimental and numerical investigation on the thermal effectiveness of metal foam with nanofluid channeling. γ-Al_2_O_3_ nanoparticles suspended in water were used as the nanofluid. The results showed that porously filled channels have increased convective strength; however, they result in a higher pressure drop [27].

Madeeha et al. [28] studied performance enhancement by integrating PCMs in the residential buildings of Pakistan. Sattar et al. [29] proposed an analytical model for the cell temperature, irradiance, and design absorbers. The cooling system was developed through the analytical modeling of cell temperature behavior and heat exchange between fluid and PV module. The optimized results were achieved at 31 passes with 186.713 W power output at 0.14 kg/s mass flow rate and 37.81 °C cell temperature. Raja et al. [30] used fuzzy multi-criteria decision-making techniques augmented with a fuzzy multi-objective mixed-integer non-linear programming mathematical model to address SSSOA problems for renewable equipment. Butt et al. [31] developed a technique involving the coupling of an oxyhydrogen (HHO) electrolyser to make the electrolyser independent of other energy sources and work as a standalone system on solar PV. The results showed 94% efficiency; additionally, the DC-DC converter played a crucial role in the system. Farooq et al. [32] explored the influence of nanodiamonds (ND) on the thermal and ablative performance of carbon-fiber-reinforced epoxy matrix composites. Their research work reveals that the addition of NDs improved weight loss due to an increased heat-affected area. Sher et al. [33] studied the direct influence of dust and humidity on PV cell performance. The results showed a decrease in output when the dust quantity increased from 72.6 g to 121 g.

The literature shows that metal foam and nanofluid have been investigated individually and in combination for electronic cooling in order to tackle the heat dissipation issues in industrial and scientific applications. Most of the studies carried out have been experimental; however, several associated benefits still need to be explored. Therefore, this study uses these combinations and explores the beneficial outcomes of nanoparticles to provide better solutions for the thermal management of devices. The analysis is performed according to the variation in the porosity and pore density for the metal foam and material and concentration of nanoparticle. The base temperature, Nusselt number, heat transfer coefficient, and pressure drop are used to determine the influence of parameters on the performance of the heat sink. It may be predicted that by modifying the fin height, spacing, and fin thickness for a rectangular-finned heat sink, the cooling systems for microelectronics devices can be substantially enhanced. The layout of this paper is as follows: Section 2 discusses materials and methods, whereas the analytical approach for the k-ε model, methodology of research, and the CAD model of heat sink and metal foam are discussed in Section 3. Section 4 provides the numerical results and discussions. The experimental validation is discussed in Section 5, followed by a conclusive summary of the research and future recommendations in Section 6.

## 2. Materials and Methods

**Figure 1:**
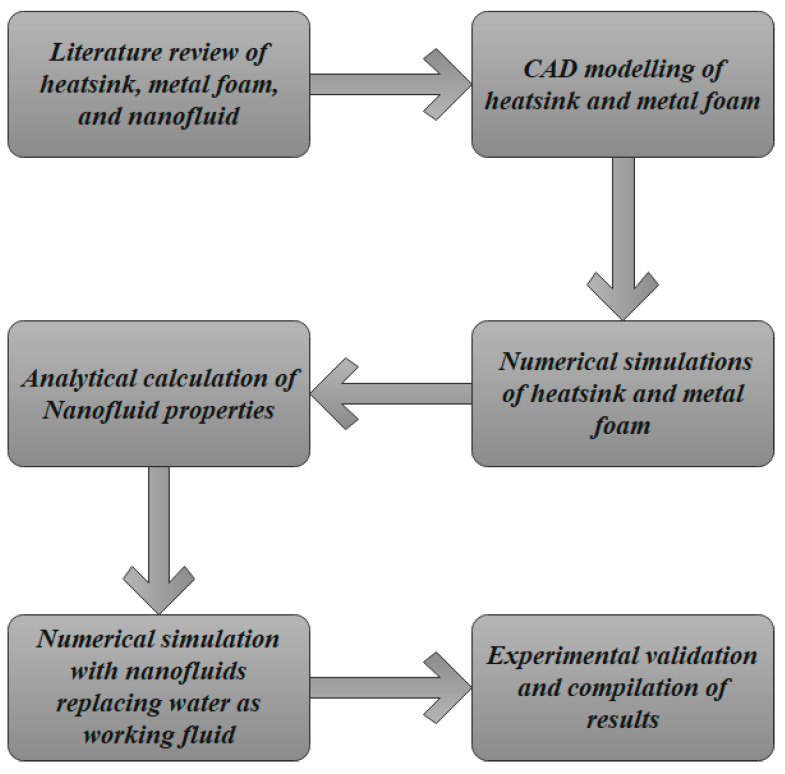
Methodology flowchart of the present research work.

The methodology followed in the present research work is depicted in Figure 1. To perform the numerical simulations, different CAD models are developed in Solid Works. Upon calculation of the y^+^ value for the range of Reynolds numbers used in the calculations, the values fell between 50 and 90. Hence, the k-ε model was used in all numerical simulations for different combinations of heat sink and metal foam with various concentrations of nanoparticles. The results acquired through numerical simulations were validated using the experimental data available in the literature.

## 3. Numerical Modeling and Simulation

The *k-ε* turbulence model is used in numerical simulations. The *k-ε* model presents two new variables into the system of equations. The continuity equation is written as follows:

(1) $$\frac{\partial \rho }{\partial t}+\frac{\partial }{{\partial x}_{i}}\;\left(\rho U_{i}\right)=0$$

The equation of momentum becomes:

(2) $$\frac{\partial }{\partial t}\;\left(\rho U_{i}\right)+\frac{\partial }{\partial x_{j}}\;\left(\rho U_{i}U_{j}\right)=-\frac{\partial \overset{´}{p}}{\partial x_{i}}+\frac{\partial }{\partial x_{j}}\;\left[\mu_{eff\;}\left(\frac{\partial U_{i}}{\partial x_{j}}+\frac{\partial U_{j}}{\partial x_{i}}\right)\right]+S_{M}$$

where $\mu_{eff\;}$ is the effective viscosity accounting for turbulence, *S_M_* is the sum of the body forces, and $\overset{´}{p}$ is the modified pressure, which is defined as follows:

(3) $$\overset{´}{p}=p+\frac{2}{3}\;\rho k+\frac{2}{3}\frac{\partial U_{k}}{\partial x_{k}}$$

The *k-ε* model is based on the concept of the eddy viscosity, therefore:

(4) $$\mu_{eff\;}=\mu_{+}\mu_{t\;}$$

where $\mu_{t\;}$ is the turbulence viscosity. The *k-ε* model considers that the turbulence viscosity is related to the turbulence kinetic energy and dissipation and the relation is as follows:

(5) $$\mu_{t\;}=C_{\mu }\rho \frac{k^{2}}{\varepsilon }C_{\mu }=0.09$$

The values of *k* and *ε* derived directly from the differential transport equations for the turbulence kinetic energy and turbulence dissipation rate are as follows:

(6) $$\frac{\partial (\rho k)}{\partial t}+\frac{\partial }{\partial x_{j}}\;\left(\rho kU_{j}\right)=\frac{\partial }{\partial x_{j}}\;\left[\left(\mu +\frac{\mu_{t}}{\sigma_{k}}\right)\;\frac{\partial k}{\partial x_{j}}\right]+P_{k}-\rho \varepsilon +P_{kb}$$

(7) $$\frac{\partial (\rho \varepsilon )}{\partial t}+\frac{\partial }{\partial x_{j}}\;\left(\rho \varepsilon U_{j}\right)=\frac{\partial }{\partial x_{j}}\;\left[\left(\mu +\frac{\mu_{t}}{\sigma_{\varepsilon }}\right)\;\frac{\partial \varepsilon }{\partial x_{j}}\right]+\frac{\varepsilon }{k}\;(C_{\varepsilon 1}P_{k}-C_{\varepsilon 2}\rho \varepsilon +C_{\varepsilon 1}P_{\varepsilon b})$$

$P_{kb}$ and $P_{\varepsilon b}$ denote the effect of the buoyancy forces. $P_{k}$ is the production turbulence due to viscous forces.

**Figure 2:**
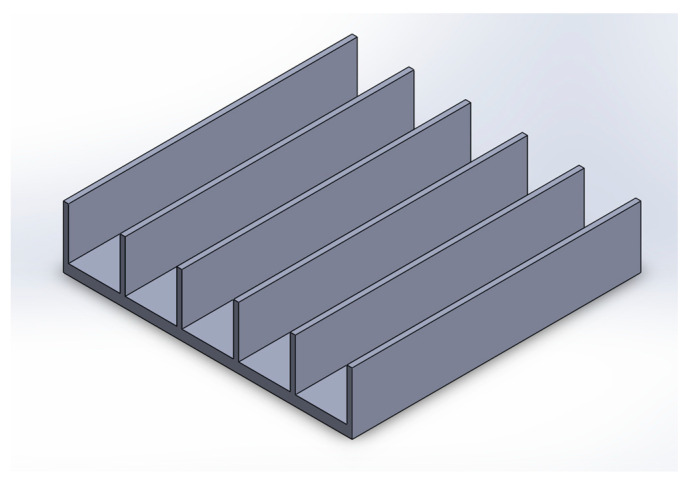
CAD model of the heat sink.

The whole setup was modeled in SOLIDWORKS 19. Different heat sinks were designed based on the variation in fin heights, base thickness, fin thickness, and fin spacing. Aluminum and copper were chosen as the heat sink material. The CAD model for the Heat sink is shown in Figure 2.

The CAD model for the metal foam is generated by applying the 3-D scanning modelling approach [34], Lord Kelvin cell [35], and the unit cell structure [36]. The 3-D scanning involves a constant value of porosity and pore density for the sample to be scanned; therefore, this approach alone cannot be used for this research. The present study requires the effects of variation in porosity and pore density and their influence on the design parameters. Moreover, the unit cell approach is chosen because it requires lower computational power and time.

**Figure 3:**
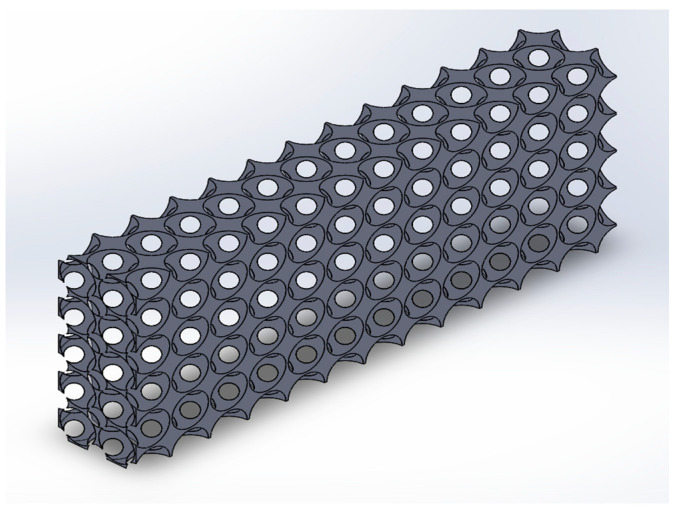
CAD model of the metal foam.

**Figure 4:**
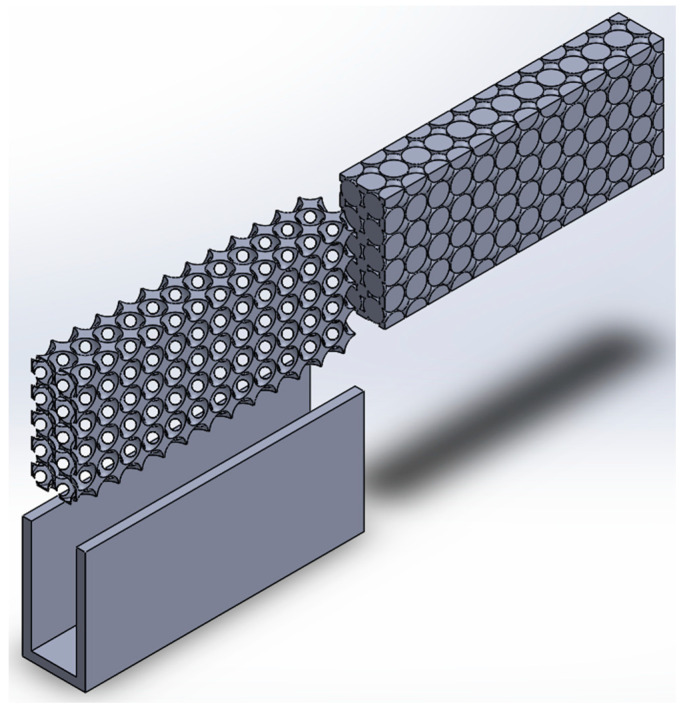
Exploded view of the assembly.

The CAD of the heat sink and metal foam are depicted in Figure 2 and Figure 3, which were designed using a BCC structure. The porosity and pore density are variables for the numerical investigation. Aluminum and copper were used to design the metal foam. The exploded view of the assembly showing the metal foam and heat sink is shown in Figure 4.

The assembly was imported to ANSYS FLUENT for further analysis. The continuity, energy, and momentum equation with the standard *k-ε* model were used as the governing equations. The equations were solved using the SIMPLE algorithm with a second-order upwind scheme.

(8) $$\nabla \left(\rho V\right)=0$$

(9) $$\left(u.\nabla \right)\rho V=-\nabla P+\mu \nabla^{2}V$$

(10) $$V.\nabla T=\frac{k}{\rho c_{p}}\nabla^{2}T$$

Water was chosen as the base fluid and a single-phase model was assumed for the nanofluid. For the nanofluids, a comparative analysis between Al_2_O_3_, Cu, CuO, SiC, and TiO_2_ with concentrations ranging between 0.3 and 1.5 % by volume was conducted. The properties of the nanofluid were determined using the following equations [17]:

- For effective density,

(11) $$\rho_{nf}=\phi \rho_{p}+\left(1-\phi \right)\rho_{w}$$

- For effective specific heat capacity,

(12) $${\left(\rho c_{p}\right)}_{nf}=\phi {\left(\rho c_{p}\right)}_{p}+(1-\phi ){\left(\rho c_{p}\right)}_{w}$$

- For effective viscosity,

(13) $$\mu_{nf}=\frac{\mu_{w}}{{\left(1-\phi \right)}^{2.5}}$$

- For effective thermal conductivity,

(14) $$k_{nf}=[\frac{k_{p}+2k_{w}-2\phi \left(k_{w}-k_{p}\right)}{k_{p}+2k_{w}+\phi \left(k_{w}-k_{p}\right)}]k_{w}$$

The boundary conditions applied for the simulations are given below.

- Heat flux on the base = 150,000 W/m^2^;
- Velocity inlet with 0.21 to 0.63 LPM flowrate;
- Pressure at the outlet = 1 atm;
- Temperature at the inlet = 300 K.

### Mesh Independence Test

**Figure 5:**
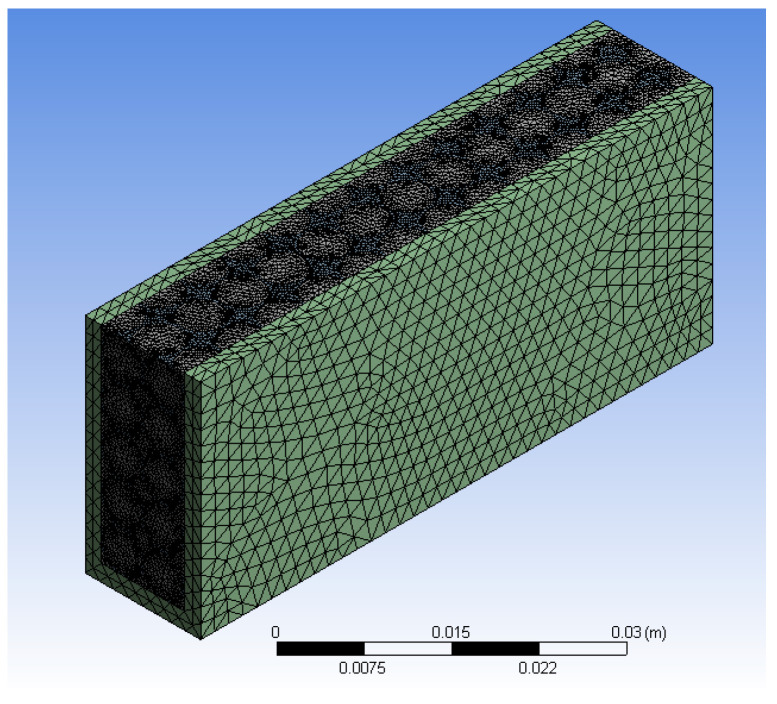
Final selected mesh model for simulations.

**Figure 6:**
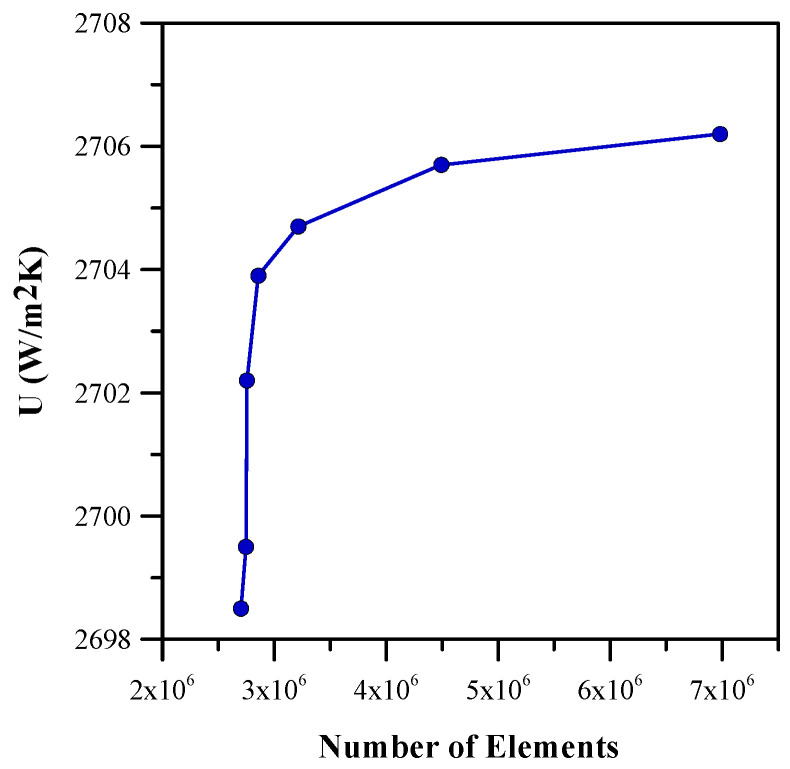
Grid independency test for heat transfer coefficient with different number of elements.

The mesh generated for the model after assembly is shown in Figure 5. A mesh independence test was performed to save computation time. Inflation was carried out with a first layer height of 0.0001, five layers, and a 1.2 growth rate. An optimum number of elements was selected to avoid time-consuming calculations and in order to obtain the correct output parameters. Figure 6 shows the mesh independence graph. The final selected mesh has almost 4.5 million elements and 917,000 nodes. The heat transfer coefficient of 2705.7 W/m^2^ was used in this study.

## 4. Results and Discussion

**Figure 7:**
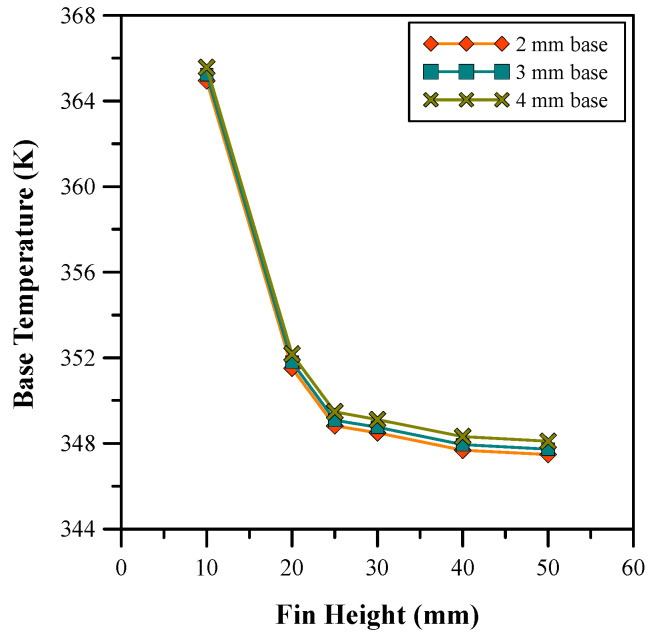
Base temperature for different fin heights.

**Figure 8:**
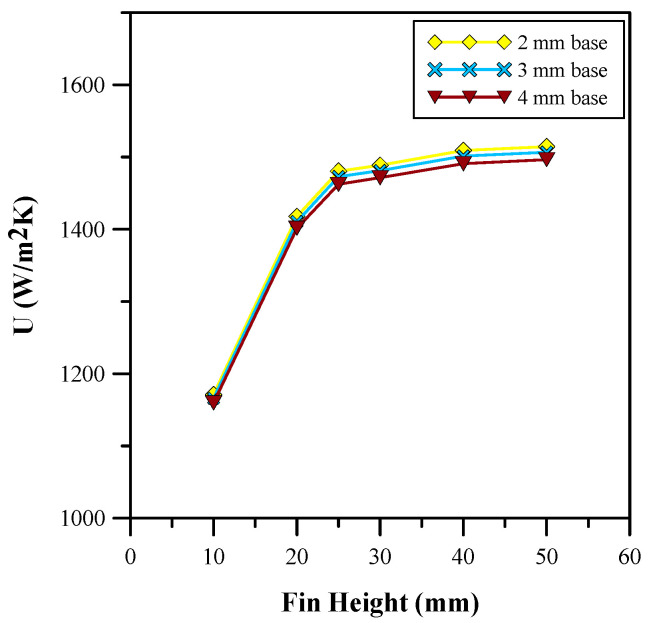
Heat transfer coefficient for different fin heights.

Figure 7 and Figure 8 show the variation in base temperature and heat transfer coefficient for different fin heights. Figure 7 depicts the base temperature decreases with an increase in fin height. This is due to an increase in the contact zone of the fluid with the surface area of the heat sink. The graph in Figure 8 shows the variation in the heat transfer coefficient for different heights of the fin. It is can be seen in Figure 8 that the heat transfer coefficient increases with the fin height, which, in turn, increases the mass flow rate of the fluid passing through the heat sink. The results shown in Figure 8 are in good agreement with the results given in [4]. Moreover, Figure 7 and Figure 8 show that a significant change occurred in the heat transfer coefficient and base temperature up to 25 mm of the fin height. However, beyond 25 mm, the values of the heat transfer coefficient become relatively constant. Furthermore, the influence of variation in the base thickness was evaluated and the optimum results were achieved with a base thickness of 2 mm. Therefore, a heat sink with a 25 mm fin height and a 2 mm base thickness was chosen for further analysis. The average base temperature and heat transfer coefficient of 348.83 K and 1480.5 W/m^2^ K was achieved for the selected parameters, respectively.

**Figure 9:**
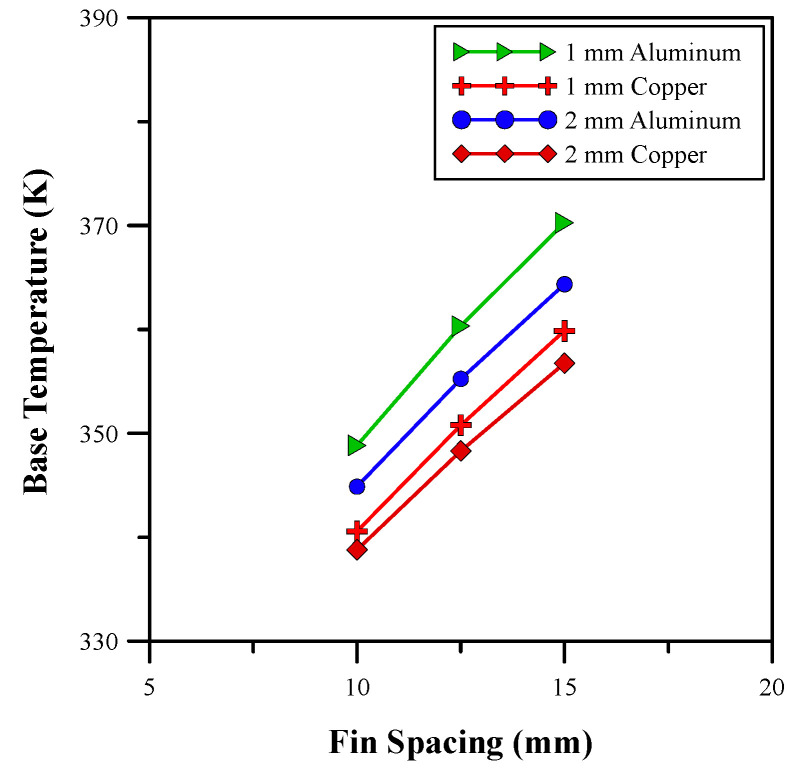
Base temperature for different fin spacings.

**Figure 10:**
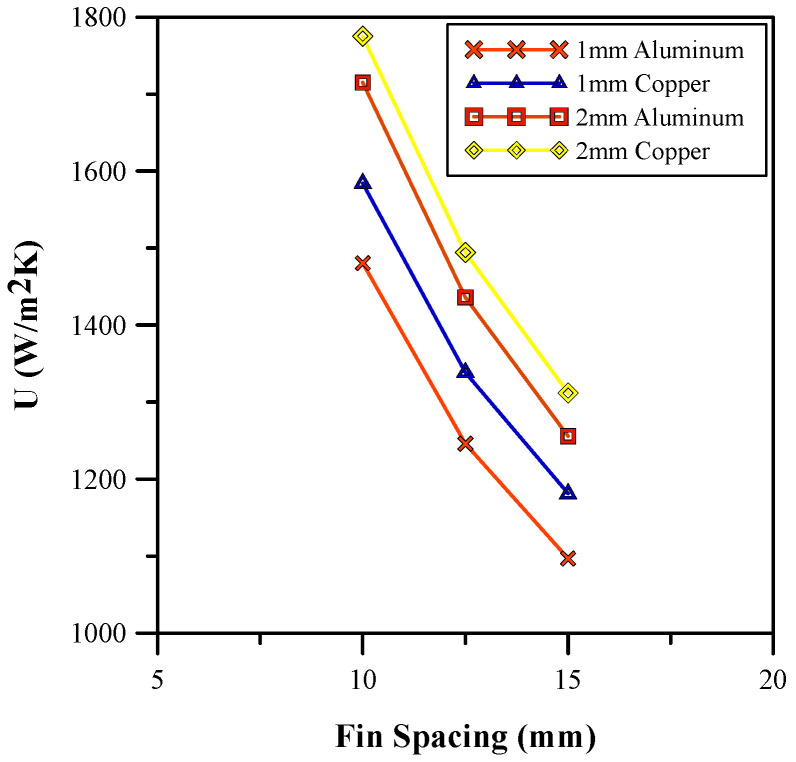
Heat transfer coefficient for different fin spacings.

After investigating the effect of different fin heights, the influence of various fin spacings on the base temperature and heat transfer coefficient is analyzed. The results of the study are depicted in Figure 9 and Figure 10, respectively. The results show that an increase in the fin spacing causes an increase in the base temperature and a decrease in the heat transfer coefficient. In [3], an optimal spacing for a heat sink at which the heat transfer coefficient is maximum is discussed. The results in Figure 9 and Figure 10 show a close resemblance with those of the research carried out by [3]. The highest heat transfer coefficient is observed for the 10 mm fin spacing. Moreover, the influence of variation in the fin thickness was evaluated and the optimum results were achieved with a fin thickness of 10 mm that results in a larger amount of heat transfer from the base. Therefore, Figure 9 and Figure 10 show a higher heat transfer coefficient and a lower base temperature. An analysis of different materials shows that copper fins showed better performance than aluminum fins due to their broader thermal boundary layer and higher rate of heat transfer [37]. Therefore, a fin spacing and thickness of 10 mm and 2 mm, respectively, was selected for further analysis of the finned heat sink design. The average base temperature was further reduced to 338.8 K and the heat transfer coefficient was improved to 1775.4 W/m^2^/K using the selected parameters. Hence, a percentage increase of 19.9% was achieved.

**Figure 11:**
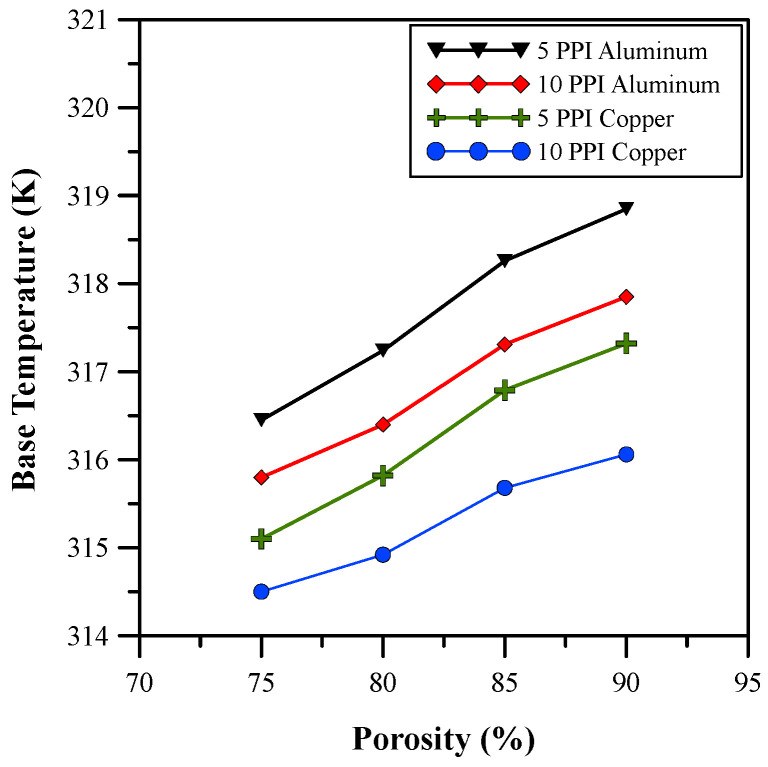
Base temperature for different porosities.

**Figure 12:**
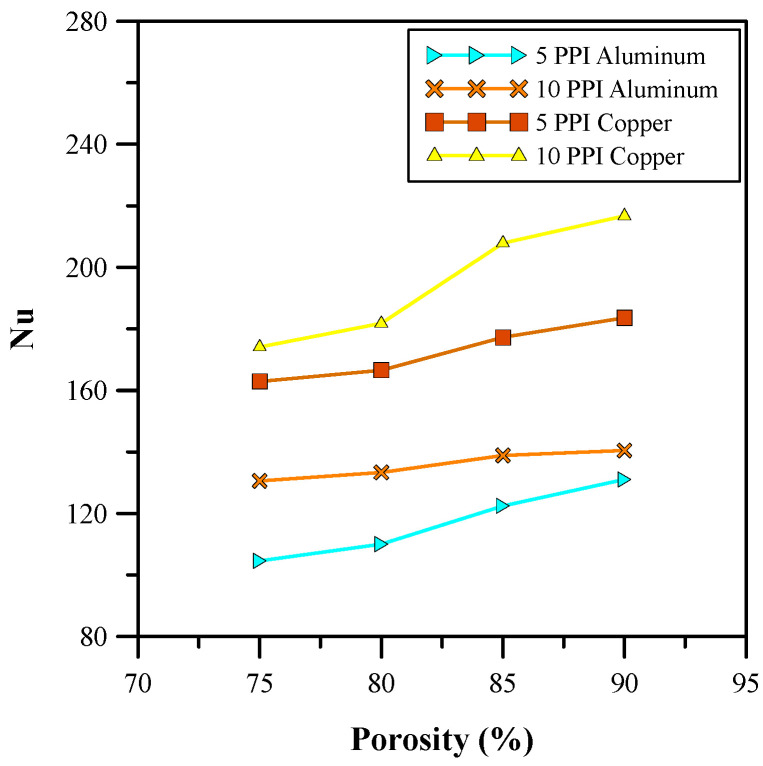
Nusselt number for different porosities.

**Figure 13:**
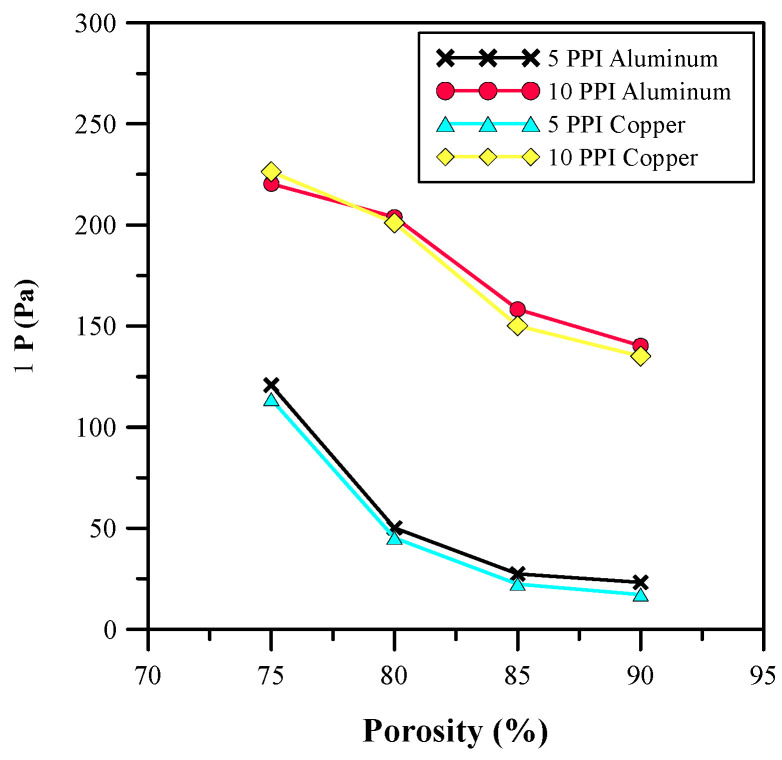
Pressure drop for different porosities.

Furthermore, the effects of the different porosities of the metal foam on the base temperature, Nusselt number, and pressure drop were studied and the results are depicted in Figure 11, Figure 12 and Figure 13. The results show that the base temperature increases with porosity and decreases with pore density. Moreover, an increase in the pore size decreases the thickness of the ligaments for the metal foam. Therefore, a drop in conduction from the metal foam to the fluid region is observed. An increase in pore density allows for a larger number of pores involved in flow mixing. Due to this, there is an increase in turbulence that minimizes the base temperature, as shown in Figure 11. Furthermore, the increase in the size of the pore allows for a higher volume of fluid flow through the pores, which increases the convective heat transfer coefficient and decreases the effective thermal conductivity of the foam. Hence, the Nusselt number increased, as shown in Figure 12, and showed an improvement of 182%. In addition to this, the average base temperature was reduced to 315.82 K through the proper adjustment of the porosity and pore density. Figure 13 shows that an increase in pore size also results in a pressure drop. The pressure drop for higher porosity and lower pore density metal foam is much higher. The analysis of variation in materials shows that copper has better thermal properties than aluminum. Therefore, copper metal foam with 85% porosity and 5 PPI was selected for further investigation.

**Figure 14:**
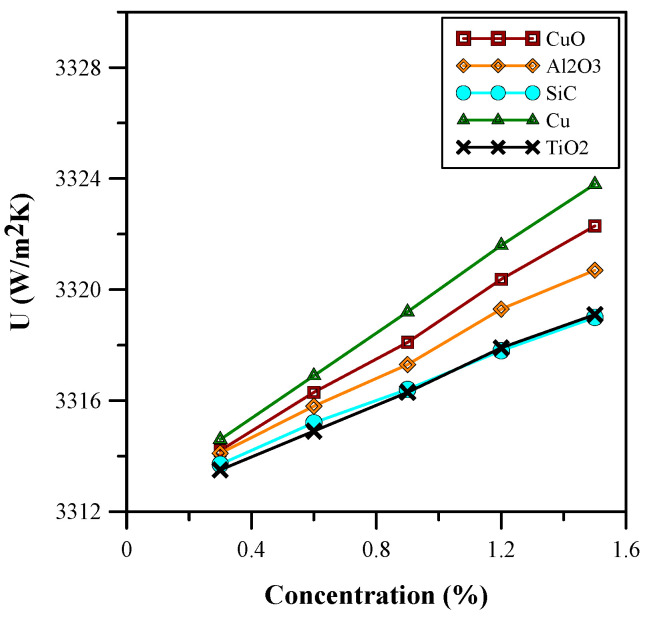
Heat transfer coefficient for varied concentrations.

**Figure 15:**
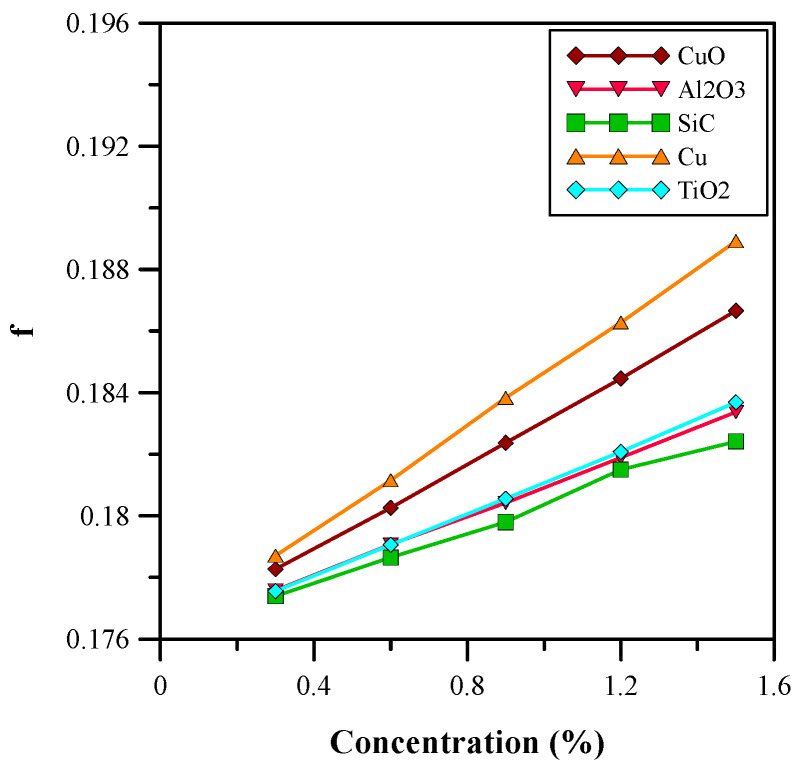
Skin Friction coefficient for varied concentrations.

Figure 14 and Figure 15 show the influence of various concentrations of nanoparticles on the heat transfer and skin friction coefficient. It is clear from Figure 13 that the heat transfer coefficient increases with the volumetric concentration of the nanofluids. The nanoparticles enhanced the thermal properties of the base fluid (water) because the nanoparticles increased the effective thermal conductivity and decreased the effective specific heat capacity. The nanoparticles also changed the effective density and viscosity of the base fluid; therefore, an increase in the skin friction coefficient was observed, as depicted in Figure 15. Nanofluids with copper nanoparticles provide the optimum results in terms of heat transfer and skin friction coefficient, and the properties of the nanoparticles are presented in Table 1. Considering the increase in pumping power, the copper nanofluid at 0.9% volumetric concentration was selected for further analysis. At a 0.9% volumetric concentration of copper, the heat transfer coefficient showed an improvement of approximately 23%.

**Figure 16:**
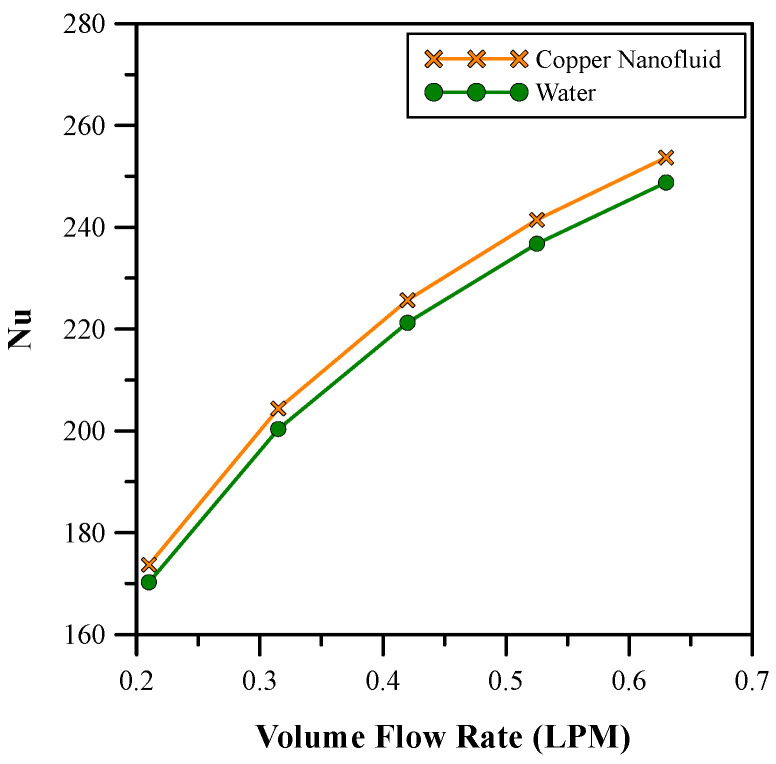
Nusselt number comparison between copper nanofluid and water.

**Figure 17:**
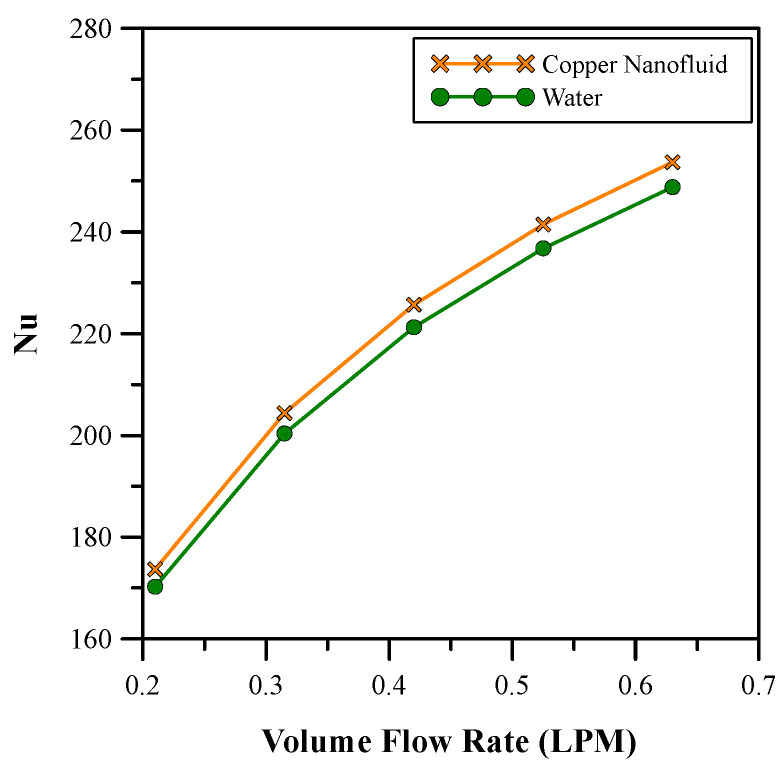
Pressure drop comparison between copper nanofluid and water.

Figure 16 and Figure 17 show the comparative analysis between the selected (0.9%) concentration of copper nanofluid with water as regards Nusselt number and pressure drop, respectively. A comparison of the results revealed that the nanofluid gave a higher Nusselt number than water for almost the same pressure drop. Pressure drop and heat transfer coefficient both increase with Reynold’s number [38], hence a balance needs to be found. The flow rate of 0.525 LPM was chosen for the nanofluid as it was the minimum flow rate with no backflow. At this flow rate, an extra pumping power of only 3.34% was required.

**Figure 18:**
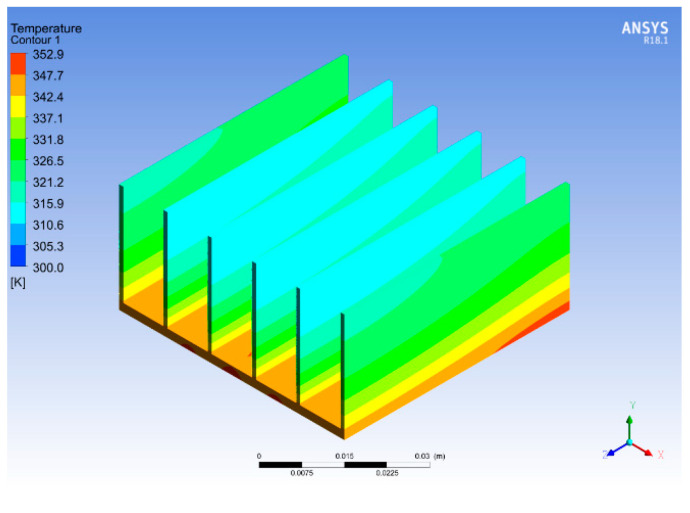
Temperature contour for varied fin heights.

**Figure 19:**
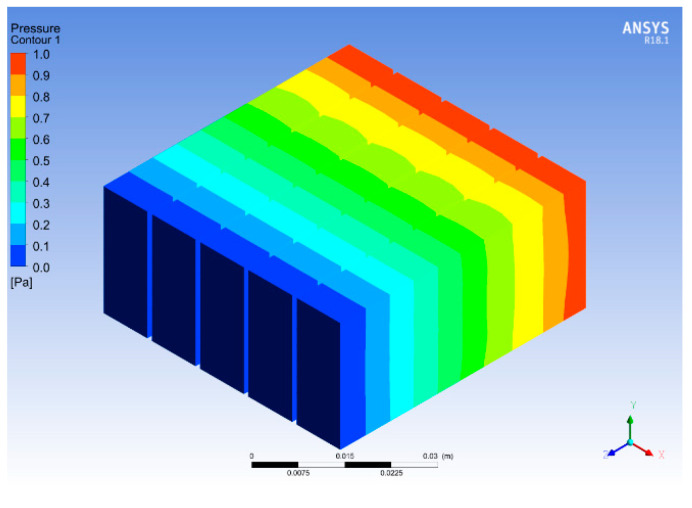
Pressure contour for varied fin heights.

Figure 18 and Figure 19 show the temperature and pressure contours for optimum fin height and base thickness. The fins enhance the surface area of the heat transfer from the base to the fluid. It can be seen that the flow of the working fluid across the heat sink results in a decreased temperature of the fins. This effect occurs due to heat transfer between the fins and the working fluid. Moreover, the temperature also decreases with an increase in fin height because a larger fin height provides a larger surface area for heat transfer. Figure 18 shows a fin height of 25 mm at which the maximum amount of heat transfer is observed. An increase in fin height beyond 25 mm results in a decrease in heat transfer enhancement. The maximum temperature of the base for the selected configuration is 352.9 K, which is higher than 323 K. In previous research, it was found that 323 K is the maximum temperature allowed for the safe operation of microelectronics; hence, further enhancement of the heat sink is required. The pressure drop across the heat sink shown in Figure 19 is due to friction between the solid and fluid surfaces. The viscosity between the heat sink and the fluid flow results in an energy loss due to the sticking effect of the fluid. The constant velocity of flowing fluid results in a pressure drop.

**Figure 20:**
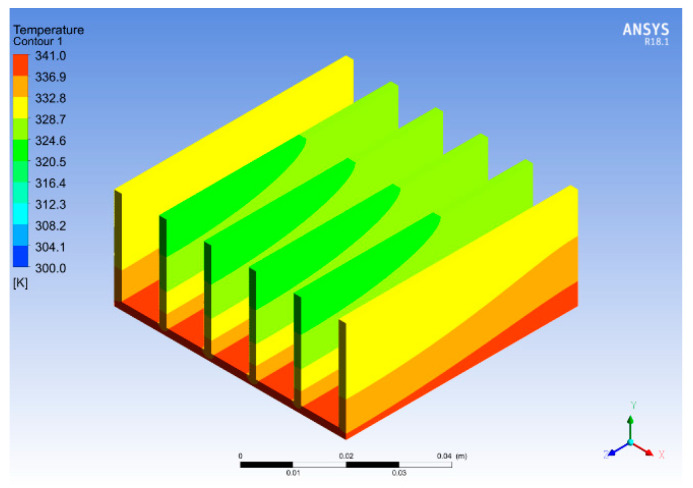
Temperature contour for varied fin spacings.

**Figure 21:**
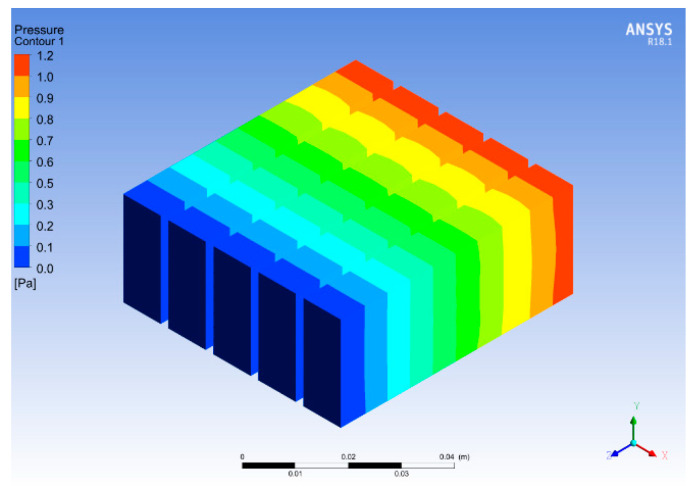
Pressure contour for varied fin spacings.

Temperature and pressure contours for varying fins spacings are shown in Figure 20 and Figure 21. The contours are obtained for 2 mm thick copper fins with 10 mm spacing. The comparison of the results demonstrated that copper, due to its better thermal conductivity, provides improved results compared to aluminum as a heat sink material. By changing the fin spacing and thickness further, the temperature of the heat sink was reduced to 341 K, which shows a 3.3% improvement in the base temperature. Moreover, the base temperature was higher at the start of the heat sink, as shown in Figure 20. The fluid flow across the heat sink dissipates the heat from the base and fins to the fluid, increasing the fluid temperature and reducing the heat sink temperature. These results are in good agreement with the research work carried out in [37], which discussed an optimum fin spacing of 12 mm in comparison to 10 mm. Furthermore, in this study, the change in geometry resulted in a pressure drop due to an increase in channel length and fin thickness, as shown in Figure 21.

**Figure 22:**
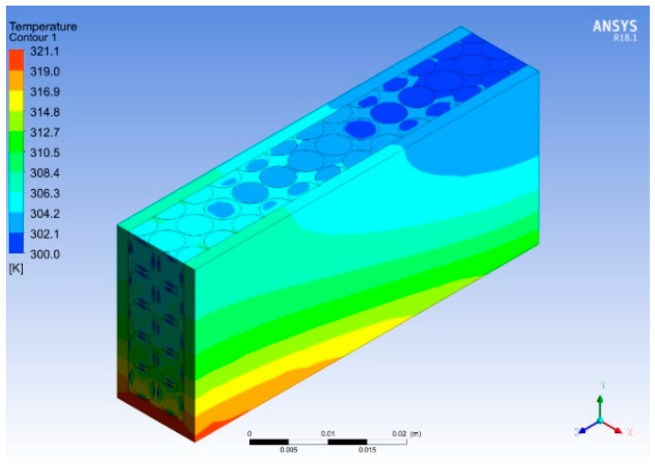
Temperature contour for varied porosities.

**Figure 23:**
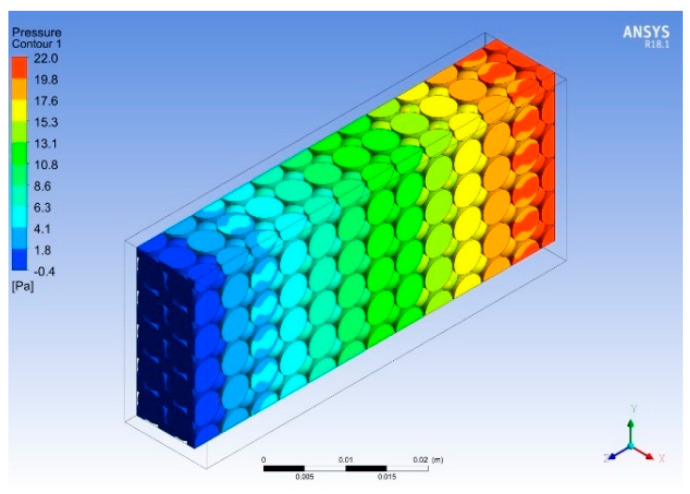
Pressure contour for varied porosities.

Figure 22 and Figure 23 show the temperature and pressure contours for varying porosities and pore densities of metal foam with the heat sink. The addition of metal foams results in a dramatic decrease in the temperature at all locations of the heat sink. The maximum temperature in the heat sink is reduced to 321 K, i.e., a percentage reduction of 5.9 is achieved, as shown in Figure 22. Moreover, the metal foam acts as a heat exchanger because it absorbs the heat from the base and effectively transfers it through the ligaments due to the higher thermal conductivity of copper. As the working fluid flows through the pores of the metal foam, more heat is absorbed by the fluid due to the turbulence and large contact surface area. The addition of cellular metal foam structure enhances the heat dissipation process and lowers the overall temperature and pressure of the heat sink, as shown in Figure 22 and Figure 23. Due to the different orientations of the pores, a mixed flow is observed as the working fluid passes through the pores, resulting in high turbulence, and increases the heat loss across the heat sink. The velocity of the flow is kept constant by overcoming the losses in the form of the loss of pressure head.

**Figure 24:**
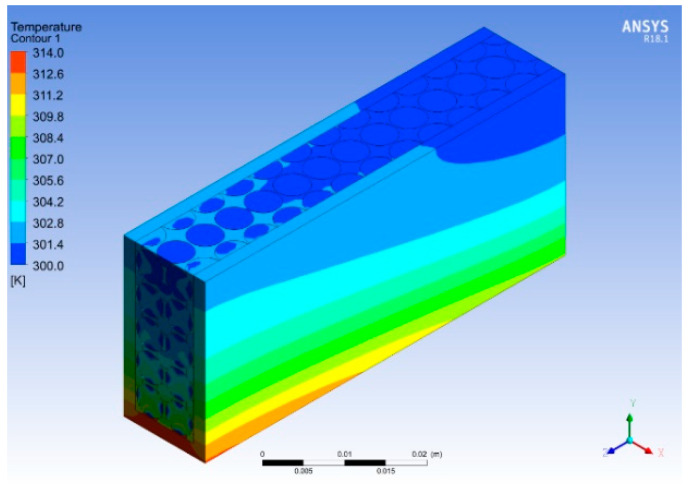
Temperature contour for varied concentrations.

**Figure 25:**
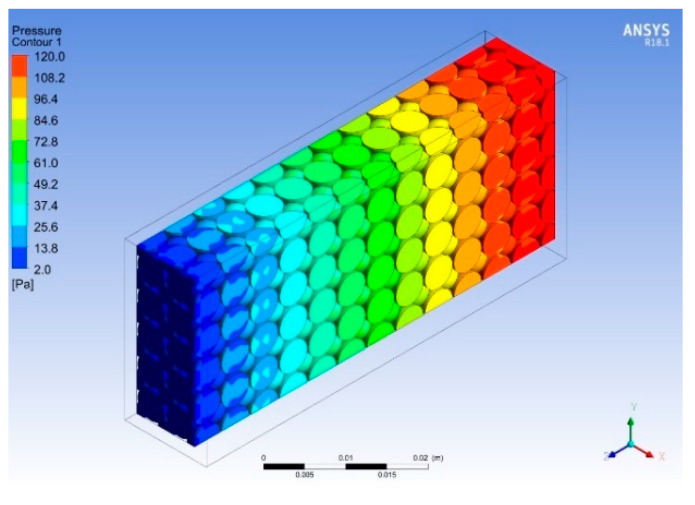
Pressure contour for varied concentrations.

Figure 24 and Figure 25 show the temperature and pressure contours for different concentrations of nanofluids. The best results were obtained for the copper nanofluid at a concentration of 9% by volume and a flow rate of 0.525 LPM. The temperature for this configuration reduced the base temperature to 314 K, thus providing a 9 K temperature drop compared to water, as shown in Figure 24. Using the nanofluid as a working fluid provides better thermal properties that result in a more effective heat transfer from the heat sink. It is observed that the highest base temperature of the heat sink at the entrance is reduced to the lowest value at the exit due to heat dissipation from the base and vice versa for the nanofluid. Moreover, the pressure is dropped across the heat sink as the Reynold’s number increases, which enhances the shear stresses and skin friction with the metal foam and finned heat sink. These results were observed to be in good agreement with those in [39]. As shown in Figure 25, the backflow observed in previous cases is eliminated when the volume flow rate reaches 0.525 LPM.

## 5. Experimental Validation

**Figure 26:**
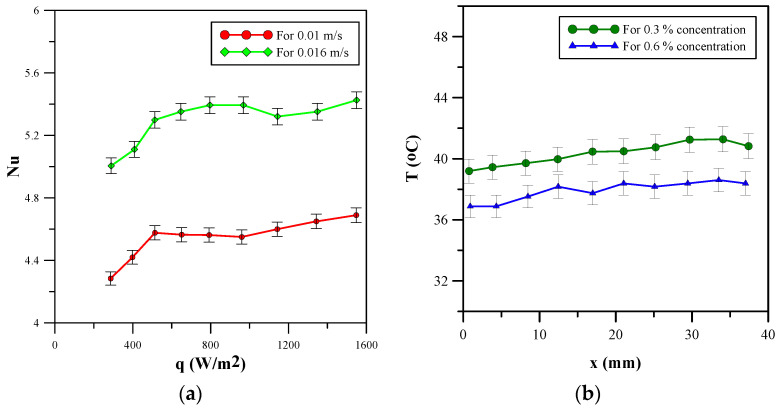
Experimental results. (**a**) Variation in Nusselt number at different fluid velocities. (**b**) Variation in base temperature with different concentrations of the nanofluid.

Firstly, the numerical results are validated with the research work of Ghaziani and Hassanipour [23]. The same procedure and boundary conditions are applied to validate the numerical simulation results. The research carried out in [23] investigated the Nusselt number, using alumina nanofluid, for the pore density ranging from 10 PPI to 40 PPI at fluid velocities of 0.01 m/s and 0.016 m/s. Figure 26a shows the variation in Nusselt number with change in the flux at different fluid velocities, i.e., 0.01 m/s and 0.016 m/s. The numerical investigation has been carried out under similar conditions for pore density at 10 PPI. Hence, the experimental results at the aforementioned pore density for 0.01 m/s and 0.016 m/s have been used for validation. The Nusselt number shows an increase with an increase in the heat flux, as depicted in Figure 26a. The figure shows that there is a decrease at 1000 W/m^2^ for the 0.01 m/s velocity and at 1200 W/m^2^ for the 0.016 m/s velocity. The experimental results have been shown with error bars to indicate uncertainty in the results. Similarly, ref. [27] investigated the base temperature of the heat sink at different locations along the channels filled by metal foam for alumina nanofluid with 0.3 and 0.6 percent of volumetric concentration. Figure 26b shows that the base temperature remains relatively constant across the channel at both concentrations of the nanofluid. A slight increase in temperature is observed towards the end of the channel. Moreover, due to the better thermal properties of the working fluid, a 0.6 percent volumetric concentration provides a lower base temperature.

**Figure 27:**
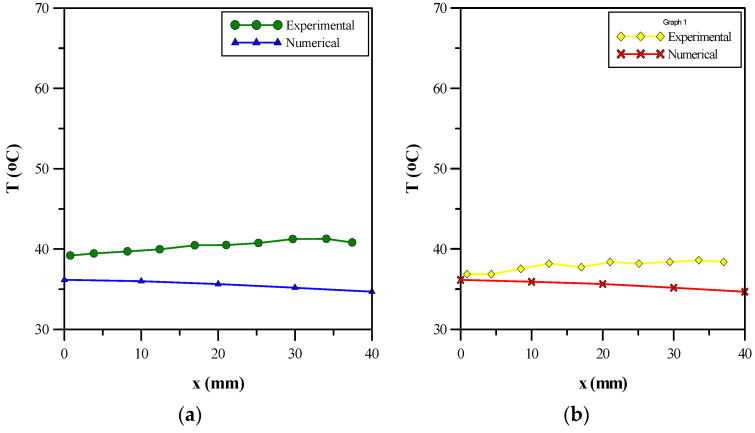
Comparison with Welsford et al. [27]. (**a**) Experimental and numerical results at 0.3 percent concentration. (**b**) Experimental and numerical results at 0.6 percent concentration.

The numerical results for the aluminum metal foam of 90% porosity were compared with the research work of [27]. The base temperature for both research works was plotted against the axial position along the heat sink. Figure 27a shows the experimental and numerical results at a volumetric concentration of 0.3%. The base temperature decreased slightly for the numerical results; however, a fluctuation was shown in the experimental results with the base temperature, i.e., first increasing slightly before decreasing and showing a relatively constant base temperature. Similarly, in Figure 27b, at 0.6% volumetric concentration, the numerical results showed a slight decrease, whereas the experimental results showed a fluctuation around a constant value for the base temperature. It can be deduced from Figure 27a,b that the numerical and experimental results are in close agreement with each other. A maximum error of 18% for a volumetric concentration of 0.3%, and 10.2% for a volumetric concentration of 0.6% between the experimental and numerical results is achieved, which is within the limits of accuracy.

**Figure 28:**
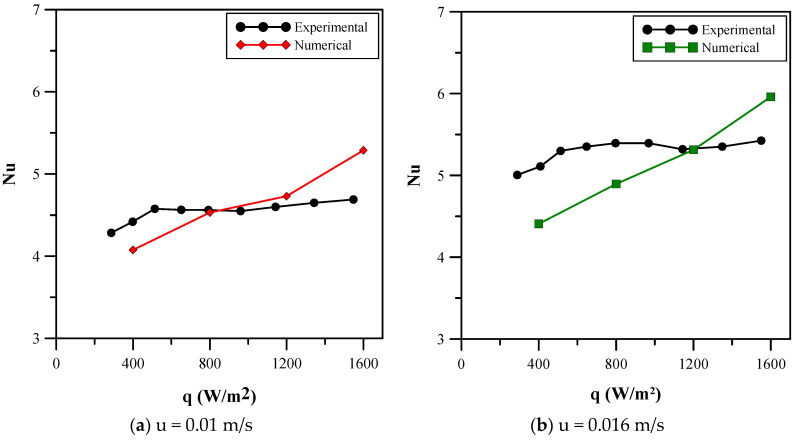
Comparison of numerical results with the experimental results of Ghaziani and Hassanipour [23]. (**a**) Analysis at 0.01 m/s velocity. (**b**) Analysis at 0.016 m/s velocity.

Another comparison was conducted for the Nusselt number with the research work of [23] at 0.01 m/s and 0.016 m/s velocity at different heat fluxes. Aluminum foam with alumina nanofluid at 0.83% volumetric concentration was used. Figure 28a shows the Nusselt number for the velocity of 0.01 m/s. It is observed from the numerical results that the Nusselt number increased with an increase in the heat flux. Initially, in the experimental results, there was an increase in the heat flux but after 600 W/m^2^, the values became relatively constant with a small amount of fluctuation. Figure 28b shows the Nusselt number for a velocity of 0.016 m/s. It can be observed that the numerical results showed an increase in the Nusselt number with increasing heat flux. The experimental results, however, fluctuate, first with an increase until a heat flux of 900 W/m^2^ is reached, followed by a decrease before attaining a relatively constant value. A comparison of the experimental and numerical results from Figure 28a,b shows that the error in the results is within the limits of accuracy. The error percent is 11.15 at 0.01 m/s velocity and 15.93 at 0.016 m/s velocity.

Elnaggar [2] experimentally investigated the heat removal rate for finned heat pipes used in cooling applications. The heat sink measured 120 × 120 × 25 mm^3^ and removed nearly 170 W of heat. The quarter of the base area of the heat sink used in this investigation removes half the heat, giving a better overall performance than that of a finned heat pipes heat sink. Jajja et al. [39] experimentally investigated mini-channel heat sinks for flow rates ranging from 0.5 to 1 LPM. A comparison of results shows that a maximum temperature drop, i.e., 50 K and 60 K, is achieved at 0.5 LPM and 0.525 LPM for the mini-channel heat sink, respectively. Gong et al. [40] numerically investigated the thermal management of electronic devices for different layouts of the micro-channel heat sink. Their research revealed that the minimum pressure drop achieved in the different layouts was nearly 250 Pa at 0.09 LPM compared to the pressure drop of 130 Pa at 0.525 LPM in this investigation. The maximum Nusselt number for the microchannel heat sinks was approximately 9.5 at 0.27 LPM, compared to 170.28 at 0.21 LPM. Thus, it is concluded that the heat sink designed in this investigation shows a better performance than that achieved when using conventional methods.

## 6. Conclusions

The use of electronic devices in numerous industries has seen a rapid increase in recent times, most notably with smart devices using microprocessors, which are currently used in devices ranging from handheld mobile phones to cars and refrigerators. Due to the working operation requirements of these devices, the heat dissipated leads to overheating and the failure of electronic devices. As a result, thermal management has become an essential step in the field of electronics’ cooling. Conventional cooling systems demand further enhancements to sustain these cooling requirements. Therefore, this research proposes novel methods to find the optimized parameters for a rectangular-finned heat sink. The sink is integrated with metal foam between the adjacent fins, and water-based nanofluids flow through the structure. ANSYS FLUENT is used for all numerical simulations. The following are the key findings of this research work.

- The current research work revealed the optimum fin height, fin spacing, base and fin thickness, and material for the heat sink. Moreover, the porosity, pore density, and material for the metal foam, and the concentration, velocity, and nanoparticle material are also investigated for the nanofluids.
- The configuration provides optimum results at a base area of 0.062 × 0.062 m^2^ and 0.025 m fin height. A base temperature of 314 K is achieved, compared to 341 K without metal foam, with a pressure drop of 130 Pa.
- Metal foam has better thermal properties and a much lower relative density compared to its solid metal counterparts.
- The three-dimensional porous structure provides flow mixing and turbulence that further enhances the performance of the finned heat sink.
- The addition of nanofluids provides further heat dissipation to the arrangement; however, the pressure drop and, in turn, the pumping power remain relatively unchanged.

Experimental validation was carried out to compare the numerical results with the experimental data from the literature. The comparison of the results from both techniques revealed acceptable amounts of error between both methods.

## Figures and Tables

**Table 1:** Nanoparticle properties.

Nanoparticle	Density (kg/m^3^)	Cp (J/kg K)	k (W/m^2^ K)
Al_2_O_3_	3970	765	46
Cu	8300	420	401
CuO	6500	535.6	20
SiC	3160	675	490
TiO_2_	4157	710	8.4

## Data Availability

Not applicable.

## Nomenclature

Al_2_O_3_	Aluminum oxide/alumina
Cu	Copper
CuO	Copper oxide
SiC	Silicon carbide
TiO_2_	Titanium oxide/titania
PPI	Pores per inch
BCC	Body-centered cubic
CAD	Computer-aided design
Re	Reynold’s number
Pr	Prandtl number
Nu	Nusselt number
Φ	Volumetric concentration
ρ_w_	Density of water
ρ_p_	Density of nanoparticles
ρ_nf_	Effective density of nanofluid
c_p,w_	Specific heat capacity of water
c_p,p_	Specific heat capacity of nanoparticles
c_p_,_nf_	Effective specific heat capacity of nanofluid
k_w_	Thermal conductivity of water
k_p_	Thermal conductivity of nanoparticles
k_nf_	Effective thermal conductivity of nanofluid
μ_w_	Viscosity of water
μ_nf_	Effective viscosity of nanofluid
U	Heat transfer coefficient
ΔP	Pressure drop
f	Skin friction coefficient
LPM	Flow rate in liters per minute
u	Velocity in x-direction
v	Velocity in y-direction
w	Velocity in z-direction
e_ij’_	Fluctuating component of rate of deformation tensor
LTE	Local thermal equilibrium
LTNE	Local thermal non-equilibrium
RNG	Renormalization group
y^+^	Dimensionless parameter for grid resolution
